# Time-reversal even charge hall effect from twisted interface coupling

**DOI:** 10.1038/s41467-023-37644-0

**Published:** 2023-04-07

**Authors:** Dawei Zhai, Cong Chen, Cong Xiao, Wang Yao

**Affiliations:** 1 Department of Physics, The University of Hong Kong, Hong Kong, China; 2 HKU-UCAS Joint Institute of Theoretical and Computational Physics at Hong Kong, Hong Kong, China

**Keywords:** Surfaces, interfaces and thin films, Electronic properties and materials

## Abstract

Under time-reversal symmetry, a linear charge Hall response is usually deemed to be forbidden by the Onsager relation. In this work, we discover a scenario for realizing a time-reversal even linear charge Hall effect in a non-isolated two-dimensional crystal allowed by time reversal symmetry. The restriction by Onsager relation is lifted by interfacial coupling with an adjacent layer, where the overall chiral symmetry requirement is fulfilled by a twisted stacking. We reveal the underlying band geometric quantity as the momentum-space vorticity of layer current. The effect is demonstrated in twisted bilayer graphene and twisted homobilayer transition metal dichalcogenides with a wide range of twist angles, which exhibit giant Hall ratios under experimentally practical conditions, with gate voltage controlled on-off switch. This work reveals intriguing Hall physics in chiral structures, and opens up a research direction of layertronics that exploits the quantum nature of layer degree of freedom to uncover exciting effects.

## Abstract (web summary)

A linear Hall response in isolated systems with time reversal symmetry is forbidden by Onsager relations. Here the authors show that this restriction is lifted by interlayer hopping in twisted bilayers, leading to a linear charge Hall effect under time reversal symmetry.

## Introduction

**Fig. 1:**
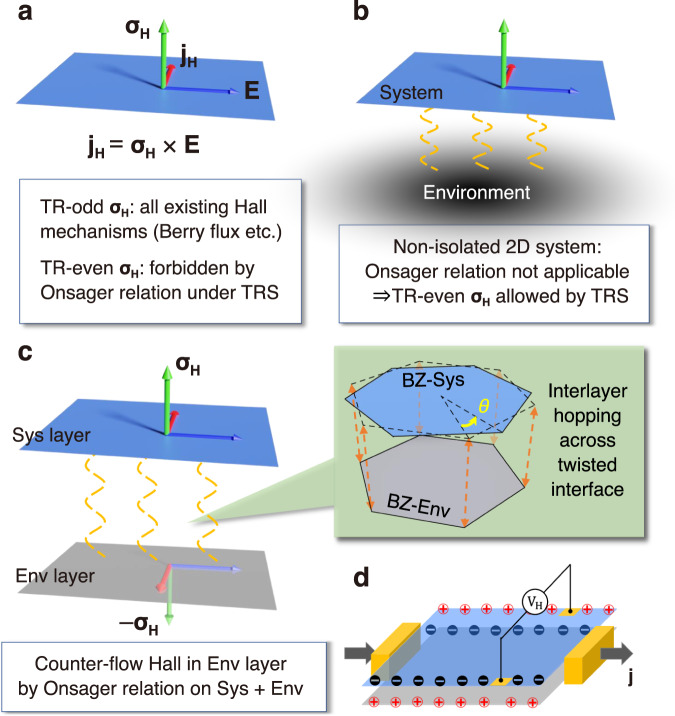
Linear charge Hall effect under time reversal (TR) symmetry in a non-isolated system. **a** In the Hall conductivity ***σ***_H_, the TR odd part by definition requires TR symmetry breaking, while the TR even part vanishes by Onsager relation. The red, green, and blue arrows denote the three vectors in **j**_H_ = ***σ***_H_ × **E**. **b** Constraint by Onsager relation can be lifted in a non-isolated 2D system (blue surface) by coupling to an environment (black shading). **c** TR-even Hall current from twisted interfacial coupling with an environmental layer (gray surface), whereas a counterflow Hall current is expected in the latter, by Onsager relation on the whole structure: system (Sys) + environment (Env). Green shaded area denotes the interlayer hopping between the Brillouin zone (BZ) with twist angle *θ*. **d** The TR-even Hall voltage (*V*_*H*_) due to charge accumulation at the sample edges (red and black + / − ) can be detected with a layer-resolved measurement. Black arrows denote source and drain current **j**.

Hall effect, by its rigorous definition, refers to a transverse charge current **j**_H_ = ***σ***_H_ × **E**, with a unidirectional or chiral nature characterized by the Hall conductivity pseudovector ***σ***_H_^1^. In principle, ***σ***_H_ can have two parts that are odd and even respectively under time reversal (TR). The TR-odd part, such as the ordinary Hall effect induced by Lorentz force and the anomalous Hall effect induced by the momentum space Berry curvature (Fig. 1a), is obviously forbidden in TR symmetric systems. The TR-even part is also forbidden under TR symmetry, with a more delicate origin in the Onsager relation of electrical conductivity^1,2^. Therefore, the linear-response charge Hall transport has been observed only in materials with TR breaking by magnetic field or magnetic order^1,3,4^.

For a non-isolated system, however, the Onsager relation on electrical conductivity is not necessarily applicable^2^, depending on the form of its interplay with the environment. This in fact leaves room for the TR-even contribution to ***σ***_H_ in the system, and hence the possibility of having charge Hall effect under TR symmetry (Fig. 1b). If the system is geometrically separated from the environment for the Hall voltage to be measurable, the TR-even Hall effect can have real impact, besides being fundamentally intriguing.

A platform to explore the above scenario is naturally provided in twisted van der Waals (vdW) layered structures^5–11^, where a two-dimensional (2D) crystal is separated by the vdW gap from adjacent layers constituting its environment (Fig. 1c). With the demonstrated capability to access electrical conduction in individual layers of the vdW structures^12–17^, this definition of system and environment becomes physical. The restriction by Onsager relation on the system layer’s conduction is lifted by quantum tunneling of electrons across the vdW gap. Without magnetic field or magnetic order, nevertheless, chiral structural symmetry is required instead to comply with the chiral nature of Hall current. This is fulfilled in a twisted stacking that breaks inversion and all mirror symmetries^18^. The intriguing TR-even Hall effect, nonetheless, remains unexplored.

In this work, we demonstrate the TR-even charge Hall effect in a twisted double layer with TR symmetry. Hall transport and charge accumulations at edges are made possible in an individual layer. Meanwhile, the Onsager relation for the whole double-layer geometry demands an opposite Hall flow in the environment layer (Fig. 1c). We find that the TR-even Hall response here is rooted in a band geometric quantity—the momentum space vorticity of layer current—that emerges from the interlayer hybridization of electronic states under TR and chiral symmetry. Our symmetry analyses show that the effect is characteristic of general chiral bilayers with Fermi surface, which we quantitatively demonstrate for the exemplary systems of twisted bilayer graphene (tBG) and twisted homobilayer transition metal dichalcogenides (tTMDs) with a wide range of twist angles. Within experimentally feasible range of carrier doping^19,20^, we find pronounced Hall responses accompanied by giant Hall ratios (e.g., ${{{{{{{\mathcal{O}}}}}}}}(1)$ in tBG), with sign and magnitude controlled by the twist angle. The effects in tTMDs also feature good on-off switchability by gate voltage that promises device applications.

## Results

### Symmetry characters

The charge Hall counterflow in system and environment layers leads to accumulation of interlayer charge dipoles (i.e., opposite charges in the two layers) near the transverse edges (Fig. 1d). Accordingly, it can be holistically viewed as the Hall transport of the charge dipole moment. The dipole Hall current measures the difference of the Hall flows in the two layers, i.e., ${{{{{{{{\bf{j}}}}}}}}}_{{{{{{{{\rm{H}}}}}}}}}^{d}={{{{{{{{\bf{j}}}}}}}}}_{{{{{{{{\rm{H}}}}}}}}}^{{{{{{{{\rm{sys}}}}}}}}}-{{{{{{{{\bf{j}}}}}}}}}_{{{{{{{{\rm{H}}}}}}}}}^{{{{{{{{\rm{env}}}}}}}}}$. Onsager relation forbids a net charge Hall current counting both layers, ${{{{{{{{\bf{j}}}}}}}}}_{{{{{{{{\rm{H}}}}}}}}}^{{{{{{{{\rm{sys}}}}}}}}}+{{{{{{{{\bf{j}}}}}}}}}_{{{{{{{{\rm{H}}}}}}}}}^{{{{{{{{\rm{env}}}}}}}}}=0$, therefore,

1 $${\sigma }_{yx}^{{{{{{{{\rm{sys}}}}}}}}}=-{\sigma }_{yx}^{{{{{{{{\rm{env}}}}}}}}}={\sigma }_{yx}^{d}/2,$$

where ${\sigma }_{yx}^{{{{{{{{\rm{sys}}}}}}}}/\,{{{{\rm{env}}}}}}$ quantifies the charge Hall effect in the system/environment layer (Fig. 1c). Such a perspective is particularly useful for identifying the symmetry requirements for the appearance of the TR-even Hall effect as elaborated in the following.

The dipole current generated at the linear order of a driving electric field **E** is given by ${j}_{a}^{d}={\sigma }_{ab}^{d}{E}_{b}$, where the Einstein summation convention is adopted for in-plane Cartesian Coordinates *a* and *b*, and ${j}_{a}^{d}$ is the current along the *a* direction of the out-of-plane interlayer dipole moment. Since the dipole current is odd under TR while the electric field is even, in nonmagnetic metallic states, the effect can only stem from nonequilibrium kinetics of electrons around the Fermi surface, and the resulting ${\sigma }_{ab}^{d}$ is a TR-even tensor. The dipole Hall conductivity, which is antisymmetric with respect to the directions of the in-plane driving field and response current, is dual to ${\sigma }_{{{{{{{{\rm{H}}}}}}}}}^{d}=({\sigma }_{yx}^{d}-{\sigma }_{xy}^{d})/2$ that transforms as a pseudoscalar (the *zz* component of a TR-even pseudotensor). Namely, it remains unchanged under rotation, but changes sign under space inversion, mirror reflection, and roto-reflection. Such a dipole Hall effect under TR symmetry is therefore allowed provided that the bilayer crystal structure is chiral.

This chiral symmetry requirement is fulfilled in the twisted bilayer vdW structures, such as tBG and tTMDs. These most studied structures, which are also our foci, are based on honeycomb lattices and preserve the threefold rotation symmetry in the *z* direction. This symmetry forbids the off-diagonal components of the symmetric part of ${\sigma }_{ab}^{d}$ with respect to *a* and *b*, i.e., ${\sigma }_{yx}^{d}+{\sigma }_{xy}^{d}=0$. Therefore, according to Eq. (1), the TR-even charge Hall current in the system layer is quantified by

2 $${\sigma }_{{{{{{{{\rm{H}}}}}}}}}^{{{{{{{{\rm{sys}}}}}}}}}={\sigma }_{{{{{{{{\rm{H}}}}}}}}}^{d}/2={\sigma }_{yx}^{{{{{{{{\rm{sys}}}}}}}}}=-{\sigma }_{xy}^{{{{{{{{\rm{sys}}}}}}}}}.$$

**Fig. 2:**
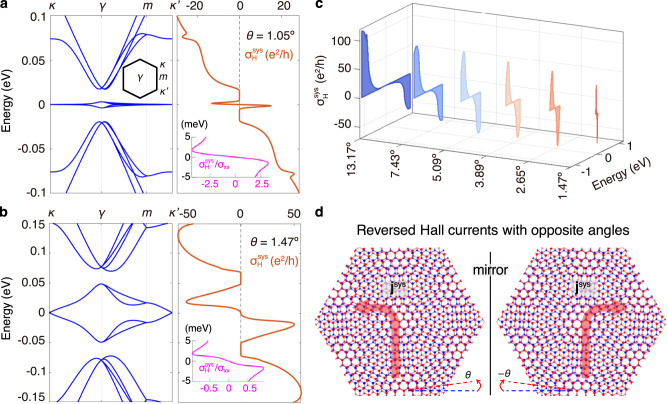
Band structure and TR-even Hall conductivity for tBG at different *θ*. **a** *θ* = 1.05^∘^. **b** *θ* = 1.47^∘^. Insets: Hall ratio of Hall conductivity ${\sigma }_{{{{{{{{\rm{H}}}}}}}}}^{{{{{{{{\rm{sys}}}}}}}}}$ to the longitudinal conductivity *σ*_*xx*_ when the Fermi level is within the central bands. **c** Evolution of the two central peaks of ${\sigma }_{{{{{{{{\rm{H}}}}}}}}}^{{{{{{{{\rm{sys}}}}}}}}}$ with *θ*. Here ${\sigma }_{{{{{{{{\rm{H}}}}}}}}}^{{{{{{{{\rm{sys}}}}}}}}}$ should be multiplied by a factor of 2 accounting for spin degeneracy. **d** Schematics of reversed Hall currents in the system layer **j**^sys^ by twisting in opposite directions, where the moiré lattices are mirror images of each other. In the calculations here and hereafter we take *τ* = 1 ps.

We point out that the direction of Hall current can be reversed with an opposite twist direction (Fig. 2d). This can be easily understood by noticing that structures obtained with twist angle *θ* and − *θ* are mirror images of each other, whereas the mirror operation flips the sign of the Hall current in each layer.

### General theory: k-space vorticity of layer current

The current density in the system and environment layer is given by the integral of the layer resolved current $e{{{{{{{{\bf{v}}}}}}}}}_{n}^{{{{{{{{\rm{sys}}}}}}}}/\,{{{{\rm{env}}}}}\,}({{{{{{{\bf{k}}}}}}}})$ carried by each electron weighted by the distribution function *f*_*n*_(**k**):

3 $${{{{{{{{\bf{j}}}}}}}}}^{{{{{{{{\rm{sys}}}}}}}}/{{{{\rm{env}}}}}}=e\mathop{\sum}\limits_{n}\int\frac{{d}^{2}{{{{{{{\bf{k}}}}}}}}}{{(2\pi )}^{2}}{f}_{n}({{{{{{{\bf{k}}}}}}}}){{{{{{{{\bf{v}}}}}}}}}_{n}^{{{{{{{{\rm{sys}}}}}}}}/\,{{{{\rm{env}}}}}\,}({{{{{{{\bf{k}}}}}}}}),$$

where *n* and *ℏ***k** are the band index and crystal momentum. ${{{{{{{{\bf{v}}}}}}}}}_{n}^{{{{{{{{\rm{sys}}}}}}}}/\,{{{{\rm{env}}}}}\,}({{{{{{{\bf{k}}}}}}}})=\langle {u}_{n}({{{{{{{\bf{k}}}}}}}})|\frac{1}{2}\{\hat{{{{{{{{\bf{v}}}}}}}}},{\hat{P}}^{{{{{{{{\rm{sys}}}}}}}}/{{{{\rm{env}}}}}}\}|{u}_{n}({{{{{{{\bf{k}}}}}}}})\rangle$, with ${\hat{P}}^{{{{{{{{\rm{sys}}}}}}}}}=(1+{\hat{l}}^{z})/2$ and ${\hat{P}}^{{{{{{{{\rm{env}}}}}}}}}=(1-{\hat{l}}^{z})/2$ being respectively the projection operator onto the system and environment layer, and ${\hat{l}}^{z}=\,{{\mbox{diag}}}\,(1,-1)$ operating in the layer index subspace^21^. Because of the TR symmetry, ${{{{{{{{\bf{v}}}}}}}}}_{n}^{{{{{{{{\rm{sys}}}}}}}}/\,{{{{\rm{env}}}}}\,}({{{{{{{\bf{k}}}}}}}})=-{{{{{{{{\bf{v}}}}}}}}}_{n}^{{{{{{{{\rm{sys}}}}}}}}/\,{{{{\rm{env}}}}}\,}(-{{{{{{{\bf{k}}}}}}}})$, hence a nonzero layer current requires a distribution function in *k*-space that breaks the occupation symmetry at **k** and − **k**. Such an off-equilibrium distribution can be driven by an electric field and described by the Boltzmann transport equation. Within the simplest constant relaxation time approximation, the deviation from the equilibrium Fermi distribution *f*_0_ ≡ *f*_0_(*ε*_*n*_) is of a dipole structure in *k*-space: ${f}_{n}-{f}_{0}=-\frac{e}{\hslash }\tau {{{{{{{\bf{E}}}}}}}}\cdot {\partial }_{{{{{{{{\bf{k}}}}}}}}}{f}_{0}$, with *ε*_*n*_ being the band energy and *τ* the transport relaxation time. This approximation is usually taken so that the specific content of disorder often unknown does not pose a difficulty and that the band origin of the effect can be manifested^22–24^.

We focus on the TR-even charge Hall response in the system layer in the following. The Hall conductivity reads

4 $${\sigma }_{{{{{{{{\rm{H}}}}}}}}}^{{{{{{{{\rm{sys}}}}}}}}}=\frac{{e}^{2}}{\hslash }\tau {{{{{{{\mathcal{V}}}}}}}},$$

where

5 $${{{{{{{\mathcal{V}}}}}}}}=-\frac{\hslash }{2}\mathop{\sum}\limits_{n}\int\frac{{d}^{2}{{{{{{{\bf{k}}}}}}}}}{{(2\pi )}^{2}}{f}_{0}^{{\prime} }{\left[{{{{{{{{\bf{v}}}}}}}}}_{n}({{{{{{{\bf{k}}}}}}}})\times {{{{{{{{\bf{v}}}}}}}}}_{n}^{{{{{{{{\rm{sys}}}}}}}}}({{{{{{{\bf{k}}}}}}}})\right]}_{z}$$

is intrinsic to the band structure, has the dimension of frequency, and is indeed a TR-even pseudoscalar conforming to the symmetry analysis. Here ${{{{{{{{\bf{v}}}}}}}}}_{n}({{{{{{{\bf{k}}}}}}}})=\langle {u}_{n}({{{{{{{\bf{k}}}}}}}})|\hat{{{{{{{{\bf{v}}}}}}}}}|{u}_{n}({{{{{{{\bf{k}}}}}}}})\rangle={{{{{{{{\bf{v}}}}}}}}}_{n}^{{{{{{{{\rm{sys}}}}}}}}}({{{{{{{\bf{k}}}}}}}})+{{{{{{{{\bf{v}}}}}}}}}_{n}^{{{{{{{{\rm{env}}}}}}}}}({{{{{{{\bf{k}}}}}}}})$, and ${f}_{0}^{{\prime} }=\partial {f}_{0}/\partial {\varepsilon }_{n}$ implies that the TR-even Hall effect is a Fermi surface property. If interlayer coupling is absent, one has ${{{{{{{{\bf{v}}}}}}}}}_{n}^{{{{{{{{\rm{sys}}}}}}}}}({{{{{{{\bf{k}}}}}}}})={{{{{{{{\bf{v}}}}}}}}}_{n}({{{{{{{\bf{k}}}}}}}})[1+{l}_{n}^{z}({{{{{{{\bf{k}}}}}}}})]/2$, hence no TR-even Hall effect. Similarly, the Hall conductivity of the environment layer can be obtained by replacing ${{{{{{{{\bf{v}}}}}}}}}_{n}^{{{{{{{{\rm{sys}}}}}}}}}({{{{{{{\bf{k}}}}}}}})$ in ${{{{{{{\mathcal{V}}}}}}}}$ with ${{{{{{{{\bf{v}}}}}}}}}_{n}^{{{{{{{{\rm{env}}}}}}}}}({{{{{{{\bf{k}}}}}}}})$. It is clear that ${\sigma }_{{{{{{{{\rm{H}}}}}}}}}^{{{{{{{{\rm{env}}}}}}}}}=-{\sigma }_{{{{{{{{\rm{H}}}}}}}}}^{{{{{{{{\rm{sys}}}}}}}}}$ and the total charge Hall current of a bilayer geometry is indeed vanishing. The formal theory thus confirms the conclusions of the foregoing symmetry arguments.

Now we show that the TR-even Hall effect has a band origin in the *k*-space vorticity of the layer current. Via integration by parts, Eq. (5) is recast into

6 $${{{{{{{\mathcal{V}}}}}}}}=\mathop{\sum}\limits_{n}\int\frac{{d}^{2}{{{{{{{\bf{k}}}}}}}}}{{(2\pi )}^{2}}{f}_{0}\,{\omega }_{n}\left({{{{{{{\bf{k}}}}}}}}\right),$$

which measures the *k*-space vorticity of the layer current ${\omega }_{n}\left({{{{{{{\bf{k}}}}}}}}\right)=\frac{1}{2}{[{{{{{{{{\boldsymbol{\partial }}}}}}}}}_{{{{{{{{\bf{k}}}}}}}}}\times {{{{{{{{\bf{v}}}}}}}}}_{n}^{{{{{{{{\rm{sys}}}}}}}}}({{{{{{{\bf{k}}}}}}}})]}_{z}$ integrated over the occupied states. As the integral of this vorticity over any full band vanishes, only ${\omega }_{n}\left({{{{{{{\bf{k}}}}}}}}\right)$ of partially occupied bands contribute to a net TR-even Hall effect. The layer current vorticity can be expressed in an enlightening form

7 $${\omega }_{n}\left({{{{{{{\bf{k}}}}}}}}\right)=\hslash {{{{{{{\rm{Re}}}}}}}}\mathop{\sum}\limits_{{n}_{1}\ne n}\frac{{\left[{{{{{{{{\bf{v}}}}}}}}}_{n{n}_{1}}\left({{{{{{{\bf{k}}}}}}}}\right)\times {{{{{{{{\bf{v}}}}}}}}}_{{n}_{1}n}^{{{{{{{{\rm{sys}}}}}}}}}\left({{{{{{{\bf{k}}}}}}}}\right)\right]}_{z}}{{\varepsilon }_{n}\left({{{{{{{\bf{k}}}}}}}}\right)-{\varepsilon }_{{n}_{1}}\left({{{{{{{\bf{k}}}}}}}}\right)},$$

where the numerator involves interband matrix elements of total velocity and layer velocity operators. Under the TR operation, interband quantities are transformed into their complex conjugates and *ε*_*n*_(**k**) is even, with which one finds that *ω*_*n*_(**k**) is also TR-even after taking the real part. This expression of ${\omega }_{n}\left({{{{{{{\bf{k}}}}}}}}\right)$ shares a striking similarity with the well-known band geometric quantity *k*-space Berry curvature^25^: The former becomes the latter if ${{{{{{{{\bf{v}}}}}}}}}_{{n}_{1}n}^{{{{{{{{\rm{sys}}}}}}}}}$ is replaced by the *k*-space interband Berry connection ${{{{{{{{\bf{A}}}}}}}}}_{n{n}_{1}}=\langle {u}_{n}|i{\partial }_{{{{{{{{\bf{k}}}}}}}}}|{u}_{{n}_{1}}\rangle$. As such, the TR-even Hall effect, despite being described by the Boltzman transport theory, encodes the information of interband coherence, which has up to now mostly connected to intrinsic transport effects induced by various Berry-phase effects^1,25–27^, and hence should be enhanced when the Fermi level is located around band near-degeneracy regions. Despite the similarity, we stress that the layer current vorticity is fundamentally different from the *k*-space Berry curvature—The latter is TR-odd and is directly involved in the noncanonical dynamical structure of semiclassical Bloch electrons^25^, while ${\omega }_{n}\left({{{{{{{\bf{k}}}}}}}}\right)$ results from electric field-induced Fermi surface shift.

It is also interesting to note that ${\omega }_{n}\left({{{{{{{\bf{k}}}}}}}}\right)=-{{{{{{{\rm{Im}}}}}}}}{{{{{{{\mathcal{C}}}}}}}}$, where ${{{{{{{\mathcal{C}}}}}}}}={\sum }_{{n}_{1}\ne n}{({{{{{{{{\bf{A}}}}}}}}}_{n{n}_{1}}\times {{{{{{{{\bf{v}}}}}}}}}_{{n}_{1}n}^{{{{{{{{\rm{sys}}}}}}}}})}_{z}$. Being the imaginary part of ${{{{{{{\mathcal{C}}}}}}}}$, the *k*-space vorticity of the layer current is connected to the real-space circulation of this current around the electron wave-packet center **r**_*c*_^25^: $\langle (\hat{{{{{{{{\bf{r}}}}}}}}}-{{{{{{{{\bf{r}}}}}}}}}_{c})\times {\hat{{{{{{{{\bf{v}}}}}}}}}}^{{{{{{{{\rm{sys}}}}}}}}}\rangle={{{{{{{\rm{Re}}}}}}}}{{{{{{{\mathcal{C}}}}}}}}$. While ${{{{{{{\rm{Re}}}}}}}}{{{{{{{\mathcal{C}}}}}}}}$ stems from the self-rotational motion of the electron wave packet, ${\omega }_{n}({{{{{{{\bf{k}}}}}}}})=-{{{{{{{\rm{Im}}}}}}}}{{{{{{{\mathcal{C}}}}}}}}$ results from the center-of-mass motion. Their relation is analogous to the *k*-space Berry curvature and quantum metric, which are the imaginary and real parts of the quantum geometric tensor^28^.

Nonzero layer current vorticity *ω*_*n*_(**k**) and a net flux ${{{{{{{\mathcal{V}}}}}}}}$ require the quantum interlayer hybridization of electronic states, which is a characteristic property not shared by Berry curvature. First, in the absence of layer-hybridized states, *ω*_*n*_(**k**) would vanish. Two scenarios of full layer polarization leading to vanishing *ω*_*n*_(**k**) can be immediately identified: (i) If the states $|{u}_{n}\rangle$ and $|{u}_{{n}_{1}}\rangle$ involved in Eq. (7) are fully polarized in the same layer around some **k**, one gets ${{{{{{{{\bf{v}}}}}}}}}_{{n}_{1}n}^{{{{{{{{\rm{sys}}}}}}}}}({{{{{{{\bf{k}}}}}}}}) \sim {{{{{{{{\bf{v}}}}}}}}}_{{n}_{1}n}({{{{{{{\bf{k}}}}}}}})[1+{l}_{n}^{z}({{{{{{{\bf{k}}}}}}}})]/2$ and thus ${\omega }_{n}\left({{{{{{{\bf{k}}}}}}}}\right) \sim 0$. (ii) If the two states are fully polarized in different layers, then ${{{{{{{{\bf{v}}}}}}}}}_{{n}_{1}n}^{{{{{{{{\rm{sys}}}}}}}}}\left({{{{{{{\bf{k}}}}}}}}\right) \sim 0$, and hence also ${\omega }_{n}\left({{{{{{{\bf{k}}}}}}}}\right) \sim 0$. Moreover, by comparing the two forms of ${{{{{{{\mathcal{V}}}}}}}}$ in Eqs. (5) and (6), one directly sees that a finite flux of vorticity also requires interlayer hybridization—If $\left|{u}_{n}\right\rangle$ is fully layer-polarized, one has ${{{{{{{{\bf{v}}}}}}}}}_{n}^{{{{{{{{\rm{sys}}}}}}}}}({{{{{{{\bf{k}}}}}}}})\propto {{{{{{{{\bf{v}}}}}}}}}_{n}({{{{{{{\bf{k}}}}}}}})$, so ${{{{{{{{\bf{v}}}}}}}}}_{n}({{{{{{{\bf{k}}}}}}}})\times {{{{{{{{\bf{v}}}}}}}}}_{n}^{{{{{{{{\rm{sys}}}}}}}}}({{{{{{{\bf{k}}}}}}}})=0$ and ${{{{{{{\mathcal{V}}}}}}}}=0$ in Eq. (5).

### Application to tBG

We now apply our theory to tBG. For small twist angles *θ*, we employ the continuum model with parameters taken from ref. ^29^. The results are corroborated by tight-binding calculations, which are also applicable at large *θ*. All model details are presented in the Methods section and Supplementary Note 2 and 3.

The calculation results for *θ* = 1.05^∘^ and 1.47^∘^ are shown in Fig. 2a, b. The central bands around zero energy are separated from their neighbors with a global gap at such small angles. When the Fermi level intersects the central bands, ${\sigma }_{{{{{{{{\rm{H}}}}}}}}}^{{{{{{{{\rm{sys}}}}}}}}}$ shows two narrow peaks with opposite signs for electron and hole doping. When the Fermi level is located in the global gap, ${\sigma }_{{{{{{{{\rm{H}}}}}}}}}^{{{{{{{{\rm{sys}}}}}}}}}$ vanishes as a Fermi surface property. Its magnitude starts to increase again when the Fermi level is shifted outside the gap. Assuming a relaxation time of 1 ps^30–32^, the TR-even Hall conductivity can reach dozens of *e*^2^/*h* upon Fermi level shifts within 20 meV. Such slight shifts can be readily achieved by dual gates. The experimental measurement shall also be facilitated by a large Hall ratio ${\sigma }_{{{{{{{{\rm{H}}}}}}}}}^{{{{{{{{\rm{sys}}}}}}}}}/{\sigma }_{xx}$, which is independent of the relaxation time if the longitudinal charge conductivity *σ*_*xx*_ is also evaluated using the constant *τ* approximation. In the current case, *σ*_*xx*_ is strongly suppressed by the quite flat dispersion, thus the Hall ratio can be ≳ 1, as shown in the inset of Fig. 2a and b.

The TR-even Hall effect is not restricted to long-wavelength moiré lattices. When *θ* gets large, the profiles of ${\sigma }_{{{{{{{{\rm{H}}}}}}}}}^{{{{{{{{\rm{sys}}}}}}}}}$ remain similar, but the width and magnitude of its peaks increase. This is illustrated in Fig. 2c, where the central peaks of ${\sigma }_{{{{{{{{\rm{H}}}}}}}}}^{{{{{{{{\rm{sys}}}}}}}}}$ are presented for a series of *θ*. While the two peaks become more separated as *θ* increases, sizable ${\sigma }_{{{{{{{{\rm{H}}}}}}}}}^{{{{{{{{\rm{sys}}}}}}}}} \sim {{{{{{{\mathcal{O}}}}}}}}(1)\,{e}^{2}/h$ within dozens of meV around zero-energy is still achievable for a wide range of *θ*.

**Fig. 3:**
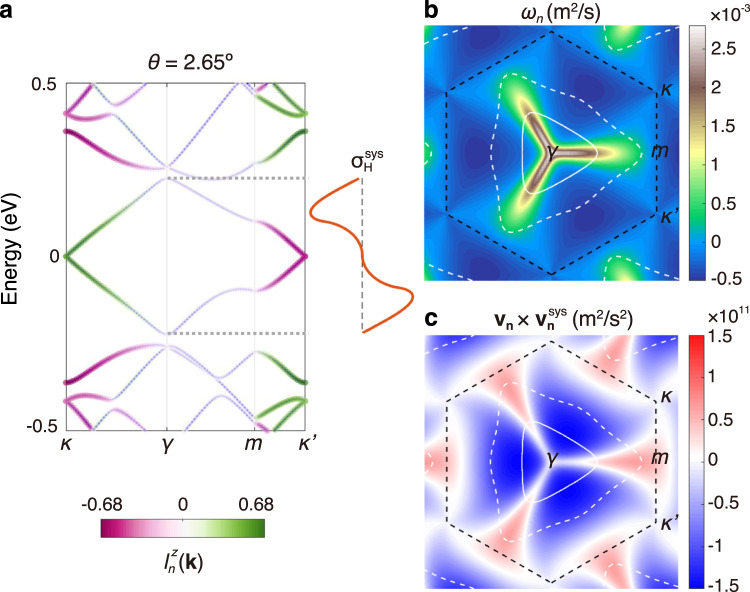
Understanding the TR-even Hall effect from band geometric quantity. **a** Band structure, **b** layer current vorticity *ω*_*n*_, and **c** ${({{{{{{{{\bf{v}}}}}}}}}_{n}\times {{{{{{{{\bf{v}}}}}}}}}_{n}^{{{{{{{{\rm{sys}}}}}}}}})}_{z}$ of the first conduction band from +K valley of 2.65^∘^ tBG. In **a**, the color coding denotes the layer composition ${l}_{n}^{z}({{{{{{{\bf{k}}}}}}}})$, and the brown curve shows the profile of central peaks of ${\sigma }_{{{{{{{{\rm{H}}}}}}}}}^{{{{{{{{\rm{sys}}}}}}}}}$. White curves in **b**, **c** show energy contours at 1/2 and 3/4 of the band width. Black dashed hexagons in **b**, **c** denote the boundary of moiré Brillouin zone.

Next we look at the *k*-space distributions of layer composition and layer current vorticity to have a better understanding of the features of the TR-even Hall effect. We illustrate these in Fig. 3 using a 2.65^∘^ tBG and focus on the first conduction band. The band projection of the layer composition ${l}_{n}^{z}({{{{{{{\bf{k}}}}}}}})$ is denoted by color in Fig. 3a. At low energies, the layer hybridization is weak, thus the states are dominantly system/environment (or top/bottom) layer polarized around *κ*/${\kappa }^{{\prime} }$. At higher energies, Dirac cones from the two layers intersect and hybridize strongly around the *γ* point, rendering ${l}_{n}^{z}({{{{{{{\bf{k}}}}}}}}) \sim 0$. Such layer polarizations or hybridizations in different band regions are also manifested in Fig. 3b for the distribution of the layer current vorticity. It is concentrated along the path from *γ* to *m*, which is characterized by regions with strongly layer-hybridized and nearly-degenerate bands, and is suppressed in the layer polarized regions around *κ* and ${\kappa }^{{\prime} }$. White curves in Fig. 3b show two different Fermi surfaces. At low electron doping, ${\sigma }_{{{{{{{{\rm{H}}}}}}}}}^{{{{{{{{\rm{sys}}}}}}}}}$ is contributed by the dark blue area in Fig. 3b with *ω*_*n*_ < 0, thus it is negative and increases in magnitude as the Fermi level is lifted towards the middle of the band [see Eq. (6) and brown curve in Fig. 3a]. As the Fermi level is further increased, regions with highly concentrated *ω*_*n*_ > 0 start to contribute, hence the magnitude of ${\sigma }_{{{{{{{{\rm{H}}}}}}}}}^{{{{{{{{\rm{sys}}}}}}}}}$ drops. Evolution of ${\sigma }_{{{{{{{{\rm{H}}}}}}}}}^{{{{{{{{\rm{sys}}}}}}}}}$ with the Fermi level can also be understood from the distribution of ${{{{{{{{\bf{v}}}}}}}}}_{n}\times {{{{{{{{\bf{v}}}}}}}}}_{n}^{{{{{{{{\rm{sys}}}}}}}}}$ shown in Fig. 3c. Since it is dominantly negative in the blue regions, according to Eq. (5), ${\sigma }_{{{{{{{{\rm{H}}}}}}}}}^{{{{{{{{\rm{sys}}}}}}}}}$ shall be negative and maximal when the Fermi level locates around the middle of the band.

In a long-wavelength moiré, the two valleys contribute to the layer Hall conductivity with identical sign and magnitude [see Eq. (7)], therefore intervalley scattering in tBG^33^ is not expected to diminish the effect. When the twist angle gets large enough, Umklapp process becomes important^34,35^, and can hybridize the two valleys and lead to new features in the layer Hall conductivity that are not expected from the continuum model. Our tight-binding calculations for *θ* = 21. 8^∘^ shows that Umklapp process leads to emergence of new conductivity peaks at low energies (see Supplementary Fig. 3).

### Application to tTMDs

**Fig. 4:**
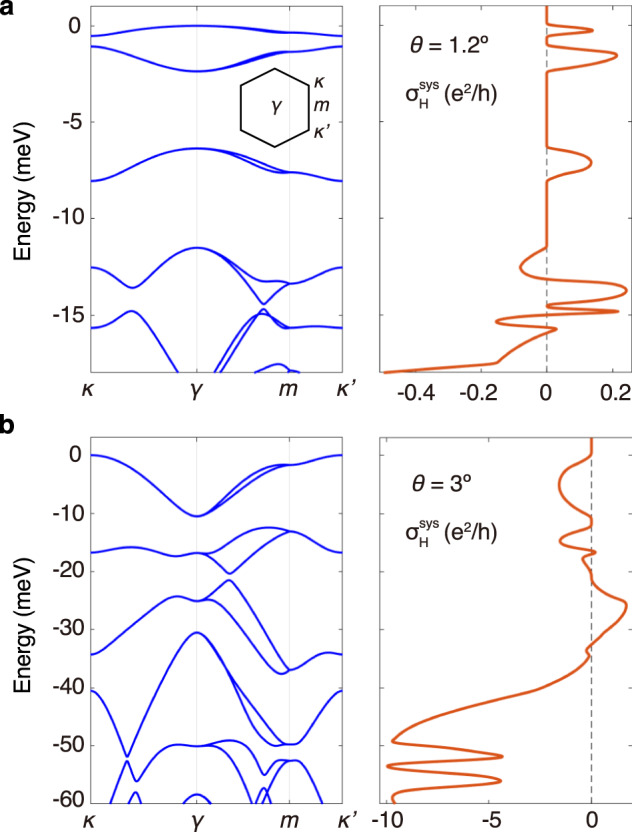
TR-even charge Hall effect in tMoTe_2_ with different *θ*. **a** Results for *θ* = 1. 2^∘^. **b** Results for *θ* = 3^∘^. In both cases, the left and right panels present the valence band structures and Hall conductivity in the system layer, respectively.

**Fig. 5:**
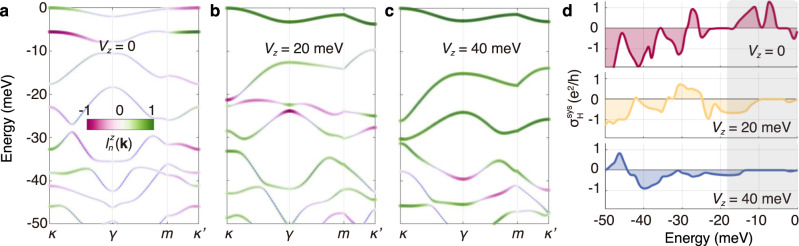
Gate control of the TR-even Hall effect in 2^∘^ tMoTe_2_. **a**–**c** Valence bands from +K valley, and **d** TR-even Hall conductivities with different interlayer bias *V*_*z*_. Color coding in **a**–**c** denotes the layer composition ${l}_{n}^{z}({{{{{{{\bf{k}}}}}}}})$. The gray shaded area in **d** highlights the gradual suppression of the TR-even Hall effect by increasing *V*_*z*_ at low energies.

Now we briefly address the TR-even Hall effect in tTMDs and focus on its tuning. We consider the continuum model of tMoTe_2_^36,37^ as an example (see Supplementary Note 1). The calculation results for *θ* = 1. 2^∘^ and 3^∘^ are shown in Fig. 4. The obtained TR-even Hall conductivity can reach ~ *e*^2^/*h* and the Hall ratio ${\sigma }_{{{{{{{{\rm{H}}}}}}}}}^{{{{{{{{\rm{sys}}}}}}}}}/{\sigma }_{xx} \sim {{{{{{{\mathcal{O}}}}}}}}(0.1)$. It exhibits rich profiles, which can be attributed to the complexity of tTMDs band structures that feature multiple isolated narrow bands, and the efficient layer hybridization in this material. As is shown in Fig. 5a for the case of *θ* = 2^∘^, most band regions are strongly layer hybridized.

Properties of tTMDs can be sensitively tuned via the interlayer bias *V*_*z*_. The band structures of 2^∘^ tMoTe_2_ with different *V*_*z*_ are shown in Fig. 5a–c, and the corresponding TR-even Hall conductivities are presented in Fig. 5d. The profiles of ${\sigma }_{{{{{{{{\rm{H}}}}}}}}}^{{{{{{{{\rm{sys}}}}}}}}}$ vary dramatically for different *V*_*z*_, including the magnitude and sign. A prominent impact of interlayer bias is the suppression of ${\sigma }_{{{{{{{{\rm{H}}}}}}}}}^{{{{{{{{\rm{sys}}}}}}}}}$ at low energies (gray area in Fig. 5d), which indicates that the TR-even Hall effect can be turned on/off with gate control. This is because the interlayer bias polarizes low-energy electrons into one of the layers (see the dominance of green color in Fig. 5b and c), thus reduces interlayer coupling.

This gate suppressed TR-even Hall effect totally differs from the gate induced layer-polarized Hall effect appearing in the even-layer antiferromagnet MnBi_2_Te_4_^38,39^. This distinction arises from the fact that the former and latter effects favor strong layer hybridization and layer polarization, respectively. It is also noted that the linear Hall effect in a nonmagnetic bilayer can only appear in the layer-counterflow manner in line with the Onsager relation (Fig. 1c), irrespective of the gate voltage; while the layer-resolved Hall effects in top and bottom ferromagnetic layers of antiferromagnetic bilayer MnBi_2_Te_4_ can be quite different when the combined symmetry of TR and space inversion is broken by the gate.

## Discussion

In summary, we have discovered the TR-even linear charge Hall effect in a non-isolated 2D crystal endowed by the twisted interfacial coupling to an environmental layer, and elucidated the band origin of this effect. Measurable effects with great tunability are predicted in paradigmatic twisted bilayer materials in the presence of TR symmetry. The layer Hall counterflow here contrasts with spin/valley Hall effect^25–27^, in the latter counterflowing Hall currents for opposite spin/valley are spatially not separated so that it is impossible to access a charge Hall current. Here, the charge Hall current in each layer is experimentally accessible with the layer-contrasted geometry in vdW devices (see Supplementary Note 5). It is also noted that the physics revealed is fundamentally distinct from the layer-polarized Hall effect in layered antiferromagnetic insulators^38–41^ and the layer-dependent quantum Hall effect^12–17^, both of which rely on the TR symmetry breaking, and from the nonlinear Hall effects^23,24,42^ where the Onsager relation is obviously irrelevant. We note that in second-order nonlinear Hall effect, there also exists a TR-even band quantity – Berry curvature dipole^42^. It, however, has different symmetry constraints from the *k*-space vorticity of layer current. In particular, it is forbidden by the threefold rotational symmetry in 2D systems, and thus is absent in tBG and tTMDs studied here. In the absence of magnetization and magnetic field, the sign of the linear Hall voltage now can be determined by the chirality of the interface, represented by the sign of the twisting angle. The effect therefore also serves as an efficient electrical probe on the structural chirality.

## Methods

### Continuum model of tBG and tTMDs

The top and bottom layers are rotated counterclockwise by ± *θ*/2, respectively, with the corresponding rotation matrix ${R}_{\pm \frac{\theta }{2}}$. In tBG, the Hamiltonian for +K valley around ${{{{{{{{\bf{K}}}}}}}}}_{0}=\left(\frac{4\pi }{3a},0\right)$ reads^29^

8 $$H=\left(\begin{array}{cc}-\hslash {v}_{F}({{{{{{{\bf{k}}}}}}}}-{{{{{{{{\bf{K}}}}}}}}}_{t})\cdot {R}_{\frac{\theta }{2}}{{{{{{{\bf{s}}}}}}}}&{{{{{{{\mathcal{U}}}}}}}}\\ {{{{{{{{\mathcal{U}}}}}}}}}^{{{{\dagger}}} }&-\hslash {v}_{F}({{{{{{{\bf{k}}}}}}}}-{{{{{{{{\bf{K}}}}}}}}}_{b})\cdot {R}_{-\frac{\theta }{2}}{{{{{{{\bf{s}}}}}}}}\end{array}\right)$$

where *a* = 2.46 Å, *v*_*F*_ = 0.8 × 10^6 ^m/s, and **s** = ( − *s*_*x*_, *s*_*y*_) with $${s}_{x}=(\begin{array}{rc}0&1\\ 1&0\end{array})$$and $${s}_{y}=(\begin{array}{rc}0&-i\\ i&0\end{array})$$the Pauli matrices in sublattice space. ${{{{{{{{\bf{K}}}}}}}}}_{t}={R}_{\frac{\theta }{2}}{{{{{{{{\bf{K}}}}}}}}}_{0}$ and ${{{{{{{{\bf{K}}}}}}}}}_{b}={R}_{-\frac{\theta }{2}}{{{{{{{{\bf{K}}}}}}}}}_{0}$ are Dirac points after rotation in the top and bottom layer, respectively. The moiré modulated interlayer tunneling is modeled by ${{{{{{{\mathcal{U}}}}}}}}={U}_{1}+{U}_{2}{e}^{-i{{{{{{{{\bf{G}}}}}}}}}_{1}\cdot {{{{{{{\bf{r}}}}}}}}}+{U}_{3}{e}^{-i({{{{{{{{\bf{G}}}}}}}}}_{1}+{{{{{{{{\bf{G}}}}}}}}}_{2})\cdot {{{{{{{\bf{r}}}}}}}}}$, where

9 $$\begin{array}{rcl}{U}_{1}&=&\left(\begin{array}{cc}{u}_{AA}&{u}_{AB}\\ {u}_{AB}&{u}_{AA}\end{array}\right)\\ {U}_{2}&=&\left(\begin{array}{cc}{u}_{AA}&{u}_{AB}{e}^{i\frac{2\pi }{3}}\\ {u}_{AB}{e}^{-i\frac{2\pi }{3}}&{u}_{AA}\end{array}\right)\\ {U}_{3}&=&\left(\begin{array}{cc}{u}_{AA}&{u}_{AB}{e}^{-i\frac{2\pi }{3}}\\ {u}_{AB}{e}^{i\frac{2\pi }{3}}&{u}_{AA}\end{array}\right)\end{array}.$$

${{{{{{{{\bf{G}}}}}}}}}_{1}=-(\frac{1}{\sqrt{3}},\,1)\frac{2\pi }{L}$ and ${{{{{{{{\bf{G}}}}}}}}}_{2}=(\frac{2}{\sqrt{3}},\,0)\frac{2\pi }{L}$ are the moiré reciprocal lattice vectors, with $L=a/(2\sin \frac{\theta }{2})$ the moiré period. The interlayer tunneling constants are *u*_*AA*_ = 79.7 meV and *u*_*AB*_ = 97.5 meV^29^. The Hamiltonian from the -K valley can be obtained from TR operation.

The continuum model of tTMDs is very similar to that of tBG, with additional electrostatic modulations in each layer^36,37^. We leave its details to the Supplementary Note 1.

### Tight-binding model of tBG

To characterize the electronic structures and TR-even Hall effect of tBG, we also use a tight-binding model following ref. ^43^. The Hamiltonian is given by

10 $${{{{{{{\mathcal{H}}}}}}}}=\mathop{\sum}\limits_{\langle i,j\rangle }t({{{{{{{{\bf{d}}}}}}}}}_{ij}){c}_{i}^{{{{\dagger}}} }{c}_{j},$$

where ${c}_{i}^{{{{\dagger}}} }$ and *c*_*j*_ are the creation and annihilation operators for the orbital on site *i* and *j*, **d**_*ij*_ represents the position vector from site *i* to *j*, and *t*(**d**_*ij*_) is the hopping amplitude between sites *i* and *j*. We adopt the following approximations

11 $$t({{{{{{{\bf{d}}}}}}}})	={V}_{pp\pi }\left[1-{\left(\frac{{{{{{{{\bf{d}}}}}}}}\cdot {{{{{{{{\bf{e}}}}}}}}}_{z}}{d}\right)}^{2}\right]+{V}_{pp\sigma }{\left(\frac{{{{{{{{\bf{d}}}}}}}}\,\cdot \,{{{{{{{{\bf{e}}}}}}}}}_{z}}{d}\right)}^{2}\\ {V}_{pp\pi }	={V}_{pp\pi }^{0}\exp \left(-\frac{d-{a}_{0}}{{\delta }_{0}}\right)\\ {V}_{pp\sigma }	={V}_{pp\sigma }^{0}\exp \left(-\frac{d-{d}_{0}}{{\delta }_{0}}\right).$$

In the above, *a*_0_ ≈ 1.42 Å is the nearest-neighbor distance on monolayer graphene, *d*_0_ ≈ 3.35 Å is the interlayer spacing, ${V}_{pp\pi }^{0}$ is the intralayer hopping energy between nearest-neighbor sites, and ${V}_{pp\sigma }^{0}$ is that between vertically stacked atoms on the two layers. Here we take ${V}_{pp\pi }^{0}=-2.7$ eV, ${V}_{pp\sigma }^{0}=0.48$ eV, *δ*_0_ is the decay length of the hopping amplitude and is set to 0.45255 Å. The hopping for *d* > 20 Åis exponentially small thus is neglected in our study.

## Supplementary information

Supplementary Information

## Data Availability

The numerical data generated by the custom codes for this study that support the findings are available from the corresponding authors on reasonable request.

## Code availability

The custom codes prepared for this study that support the findings are available from the corresponding authors on reasonable request.
