# Publisher Correction: Time-reversal even charge hall effect from twisted interface coupling

**DOI:** 10.1038/s41467-023-40030-5

**Published:** 2023-07-17

**Authors:** Dawei Zhai, Cong Chen, Cong Xiao, Wang Yao

**Affiliations:** 1 Department of Physics, The University of Hong Kong, Hong Kong, China; 2 HKU-UCAS Joint Institute of Theoretical and Computational Physics at Hong Kong, Hong Kong, China

**Keywords:** Surfaces, interfaces and thin films, Electronic properties and materials

Correction to: *Nature Communications* 10.1038/s41467-023-37644-0, published online 07 April 2023

The original PDF version of this Article contained an error in the expression for the k-space vorticity of the layer current on the second line below Equation 6, where a partial derivative was missing as the first term after the opening of the square bracket. This has been corrected in the PDF version of the Article.

