# Supplementary material for: Time-reversal even charge hall effect from twisted interface coupling
Source: Nat Commun. 2023 Apr 7;14:1961. doi: 10.1038/s41467-023-37644-0 (PMC10082030; doi:10.1038/s41467-023-37644-0)
Supplement: Supplementary file 1 — Supplementary Information [file 41467_2023_37644_MOESM1_ESM.pdf]

Supplementary Information

Time-Reversal Even Charge Hall Effect From Twisted Interface Coupling

Dawei Zhai,[1,2,*] Cong Chen,[1,2,*] Cong Xiao,[1,2,†] and Wang Yao[1,2,‡]

[1]Department of Physics, The University of Hong Kong, Hong Kong, China
[2]HKU-UCAS Joint Institute of Theoretical and Computational Physics at Hong Kong, China

Contents

Supplementary Note 1. Continuum model of near 0° twisted homobilayer TMDs 2
Supplementary Note 2. Tight-binding model results for tBG 3
Supplementary Note 3. Comparison of results from continuum and tight-binding models in tBG 4
Supplementary Note 4. Effects of Umklapp intervalley process in 21.8° tBG 4
Supplementary Note 5. Proposal of experimental setup 5

References 5

[*]These authors contributed equally to this work.
[†]Electronic address: congxiao@hku.hk
[‡]Electronic address: wangyao@hku.hk

Supplementary Note 1. Continuum model of near 0° twisted homobilayer TMDs

We assume that the top and bottom layers are rotated counterclockwise by $\pm\theta/2$ respectively with the corresponding rotation matrix $R_{\pm\frac{\theta}{2}}$. The Hamiltonian reads

$$H = \begin{pmatrix} \hbar v_F(\mathbf{k}-\mathbf{K}_t)\cdot R_{\frac{\theta}{2}}(s_x,s_y) + \mathrm{diag}(E_g,0) + \mathcal{V}_t & \mathcal{U} \\ \mathcal{U}^\dagger & \hbar v_F(\mathbf{k}-\mathbf{K}_b)\cdot R_{-\frac{\theta}{2}}(s_x,s_y) + \mathrm{diag}(E_g,0) + \mathcal{V}_b \end{pmatrix} \quad (1)$$

around $\mathbf{K}_0 = (\frac{4\pi}{3a}, 0)$ for spin up carriers. Note that here zero energy is set at valence band edge. In the following, we consider $MoTe_2$, and use the parameters $a = 3.472$ Å, $v_F = 0.4\times10^6$ m/s, monolayer band gap $E_g = 1.1$ eV [1]. The rest of the notations are consistent with those in tBG in the Methods. To incorporate effects of interlayer bias, one adds $V_z/2$ and $-V_z/2$ to the two diagonal blocks, respectively.

The electrostatic modulation in the diagonal terms of H are given by $\mathcal{V}_{l=t,b} = \begin{pmatrix} V_l^c & 0 \\ 0 & V_l^v \end{pmatrix}$ with

$$\begin{aligned} V_t^c &= V_0^c \sum_{i=1}^{3} \cos(\mathbf{G}_i\cdot\mathbf{r}+\alpha_c) \\ V_b^c &= V_0^c \sum_{i=1}^{3} \cos(\mathbf{G}_i\cdot\mathbf{r}-\alpha_c) \\ V_t^v &= V_0^v \sum_{i=1}^{3} \cos(\mathbf{G}_i\cdot\mathbf{r}+\alpha_v) \\ V_b^v &= V_0^v \sum_{i=1}^{3} \cos(\mathbf{G}_i\cdot\mathbf{r}-\alpha_v) \end{aligned}, \quad (2)$$

where $V_0^c = 11.94$ meV, $V_0^v = 16$ meV, $\alpha_c = 87.9^\circ$, and $\alpha_c = 89.6^\circ$ in the case of $MoTe_2$ [1]. Same as the case of tBG in the Methods section, $\mathbf{G}_1 = -(1/\sqrt{3}, 1)2\pi/L$, $\mathbf{G}_2 = (2/\sqrt{3}, 0)2\pi/L$, and $\mathbf{G}_3 = -\mathbf{G}_1 - \mathbf{G}_2$.

The interlayer tunneling terms in the off-diagonal terms of H are given by

$$\mathcal{U} = \begin{pmatrix} u_{cc} & u_{cv} \\ u_{vc} & u_{vv} \end{pmatrix} + \begin{pmatrix} u_{cc} & u_{cv}e^{-i\frac{2\pi}{3}} \\ u_{vc}e^{i\frac{2\pi}{3}} & u_{vv} \end{pmatrix} e^{-i\mathbf{G}_1\cdot\mathbf{r}} + \begin{pmatrix} u_{cc} & u_{cv}e^{i\frac{2\pi}{3}} \\ u_{vc}e^{-i\frac{2\pi}{3}} & u_{vv} \end{pmatrix} e^{-i(\mathbf{G}_1+\mathbf{G}_2)\cdot\mathbf{r}}, \quad (3)$$

where $u_{cc} = -2$ meV, $u_{vv} = -8.5$ meV, and $u_{cv} = u_{vc} = 15.3$ meV in the case of $MoTe_2$ [1].

We assign the Hamiltonian in the above as the +K valley in the main text. The Hamiltonian from the -K valley can be obtained from TR operation.

Supplementary Note 2. Tight-binding model results for tBG

Supplementary Figure 1 shows the band structures and corresponding TR-even Hall conductivity in tBG from tight-binding calculations.

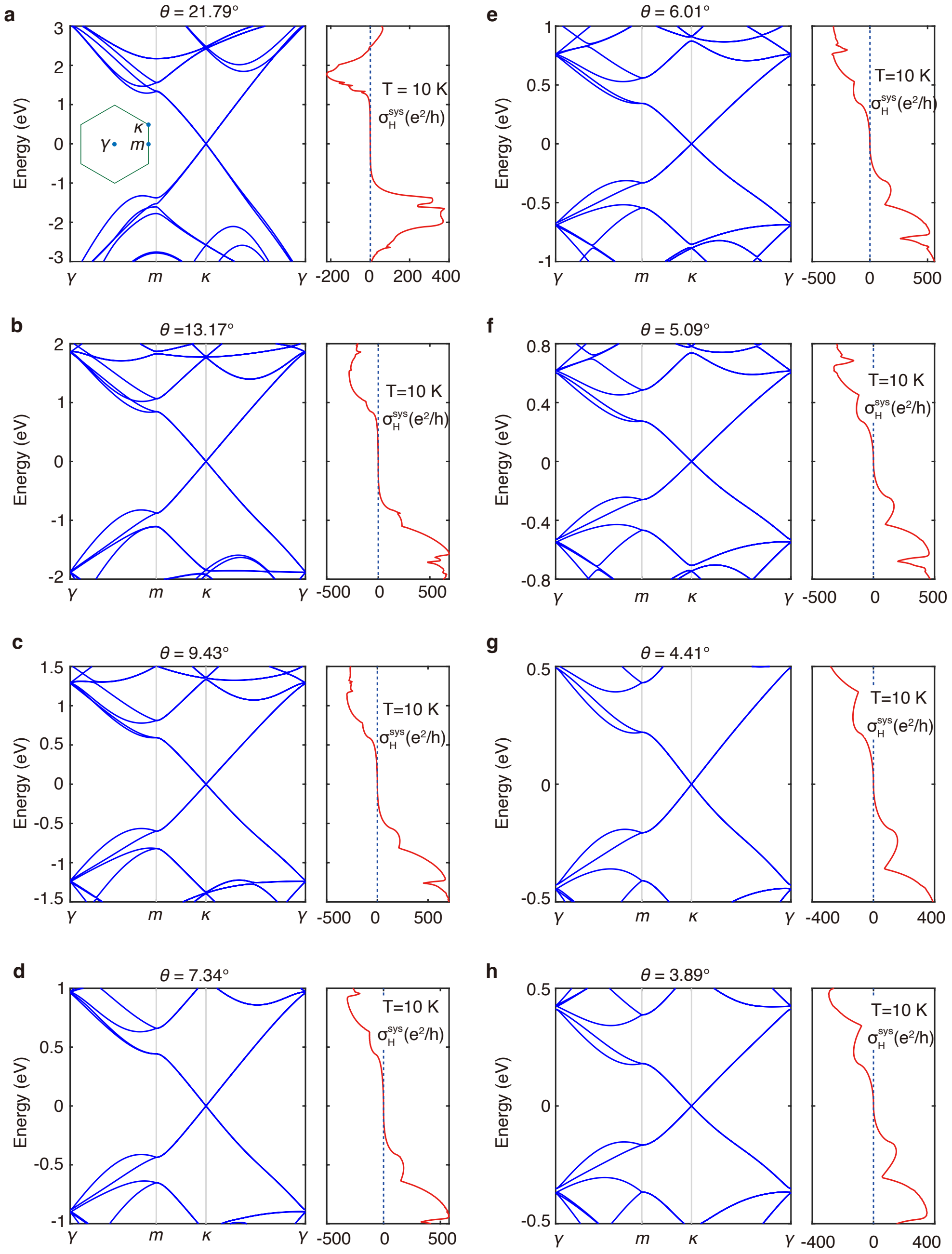

Supplementary Figure 1: **Results from tight-binding calculations.** Band structures (left) and corresponding TR-even Hall conductivity (right) of tBG with **a** $\theta = 21.79°$, **b** $\theta = 13.17°$, **c** $\theta = 9.43°$, **d** $\theta = 7.34°$, **e** $\theta = 6.01°$, **f** $\theta = 5.09°$, **g** $\theta = 4.41°$ and **h** $\theta = 3.89°$. Note that spin degeneracy is taken into account in σ_H^{sys}.

Supplementary Note 3. Comparison of results from continuum and tight-binding models in tBG

Supplementary Figure 2 shows the comparison of energy bands and TR-even Hall conductivity of 3.89° tBG obtained from continuum model and tight-binding calculations. It is clear that the two methods yield consistent results. It should be noted that we have taken into account the effect of lattice relaxation in the continuum model by setting different values for interlayer tunneling between same-atom sites and different-atom sites [2]. While such effect is not included in tight-binding calculations. Better agreement could be achieved if such effect is considered or neglected [3] in both methods.

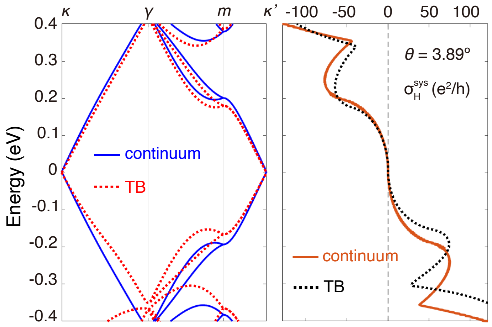

Supplementary Figure 2: Comparison of results from continuum model (solid curves) and tight-binding calculations (dashed curves). (Left) Band structure, (right) TR-even Hall conductivity of 3.89° tBG. Results of $\sigma_H^{\rm sys}$ should be multiplied by a factor of 2 taken into account spin degeneracy.

Supplementary Note 4. Effects of Umklapp intervalley process in 21.8° tBG

Umklapp process becomes prominent near the Dirac points at large commensurate twist angles (e.g., $\theta = 21.8°$) [4, 5]. To examine the effect of Umklapp process, we have performed the tight-binding calculation for tBG at $\theta = 21.8°$ (Supplementary Figure 3). The obtained energy spectrum shows gap opening at the Dirac points (Supplementary Figure 3b), which is consistent with previous results [3, 5]. Remarkably, the Hall conductivity shows new features (Supplementary Figure 3c) that are not expected from the continuum model. Note that the trend found in the small θ regime is that the conductivity peaks move to higher energy with the increase of θ and have opposite signs in the conduction and valence bands (Figure 2c of main text). At $\theta = 21.8°$, however, new conductivity peaks emerge at low energies with the same sign in the conduction and valence bands (Supplementary Figure 3c). This can be attributed to the Umklapp process.

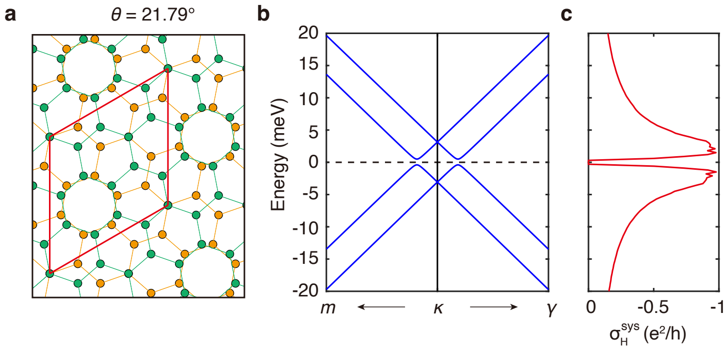

Supplementary Figure 3: **a** Moiré of $\theta = 21.8°$ tBG. The red lines enclose one unit cell. **b** Low-energy band structures near the Dirac points. **c** Hall conductivity in the system layer.

Supplementary Note 5. Proposal of experimental setup

Fabrication of electrical contacts to one individual layer for layer-resolved measurement is experimentally feasible in coupled bilayer systems. Supplementary Figure 4a shows the schematics of the experimental setup. When two monolayer flakes with different sizes/shapes are stacked, and etched into a Hall bar geometry, the overlapped part (twisted bilayer region) serves as the main channel connected to the source-drain contacts. Meanwhile, individual contacts can be deposited onto the monolayer parts of the Hall bar, which can be used as layer-resolved Hall voltage probes.

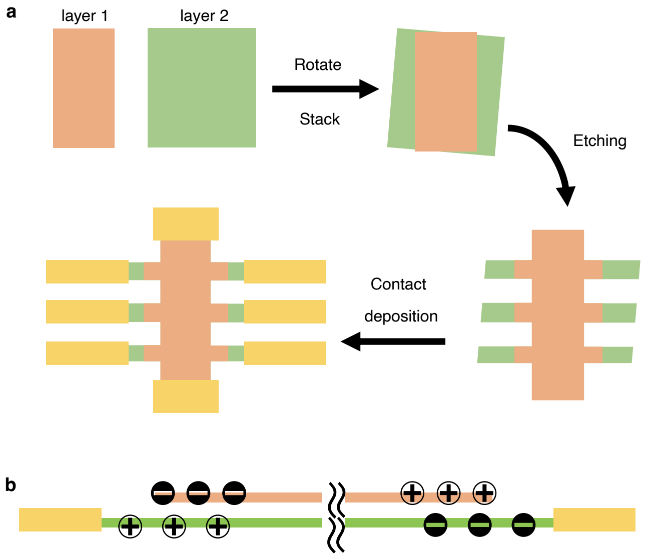

Supplementary Figure 4: **a** Stacking two monolayers with different sizes/shapes (orange and green surfaces), and etching them into a Hall bar geometry. Electrical contacts (yellow) can be deposited onto the monolayer regions of Hall bars for layer-resolved measurement in **b**.

We notice that certain quantitative experimental uncertainties may exist, but they are not expected to qualitatively affect the observation. As a result of the predicted layer contrasted Hall effect, charges accumulate on the edges with layer and edge dependent signs (Supplementary Figure 4b). The interface between the bilayer and monolayer regions on the Hall bar arms can have quantitative effect on the charge distribution in the measured layer (green layer in Supplementary Figure 4), hence the transverse voltage drop measured by the electrical probes may deviate from the value predicted by our bulk theory without considering these details. However, order-of-magnitude reduction of the predicted effect is not expected when the contacts are close to the bilayer area.

---

[1] F. Wu, T. Lovorn, E. Tutuc, I. Martin, and A. H. MacDonald, Phys. Rev. Lett. **122**, 086402 (2019).
[2] M. Koshino, N. F. Q. Yuan, T. Koretsune, M. Ochi, K. Kuroki, and L. Fu, Phys. Rev. X **8**, 031087 (2018).
[3] P. Moon and M. Koshino, Phys. Rev. B **87**, 205404 (2013).
[4] E. J. Mele, Phys. Rev. B **81**, 161405 (2010).
[5] M. J. Park, Y. Kim, G. Y. Cho, and S. Lee, Phys. Rev. Lett. **123**, 216803 (2019).
